# Directional motion of the foam carrying oils driven by the magnetic field

**DOI:** 10.1038/s41598-021-00744-2

**Published:** 2021-10-28

**Authors:** Xiaoxiao Dou, Zhewen Chen, Pingcheng Zuo, Xiaojian Cao, Jianlin Liu

**Affiliations:** College of Pipeline and Civil Engineering, China University of Petroleum (East China), Qingdao, 266580 China

**Keywords:** Energy science and technology, Nanoscience and technology, Physics

## Abstract

Foams are substances widely used the foam flooding technology, which aim to greatly improve the residual oil recovery. In the present study, we perform a comprehensive investigation on the oil removal process driven by the foam embedded with magnetic particles, under the action of the magnetic force. The experiment shows that the addition of magnetic particles has little effect on the stability of the foam. During the motion of the foam, its maximum displacement and maximum acceleration are fully explored. Such factors as the volume of the foam, the volume of the oil droplet, the mass concentration of magnetic particles, and the Young’s contact angle of surfactant on solid are surveyed in detail. The function curves of the maximum displacement and the maximum acceleration with respect to these variables are obtained in the experiment, and the selection of some optimal parameters is advised. Moreover, the dimensional analysis has been conducted and several scaling laws are given, which are in agreement with the experimental results. These findings are beneficial to understand the oil displacement with the aid of magnetic field, which also provide some inspirations on drug delivery, robots and micro-fluidics.

## Introduction

Foam is a kind of soft matter with special structures, which has the advantages of low density, strong ability to carry solid particles, little damage to the formation and easy to be discharged from the stratum^1–3^. In the petroleum engineering, foams are normally injected into the stratum under the external pressure, playing the role of the displacement medium. Due to the good plugging performance, foams are easier to block large pores, forcing the subsequent foams to penetrate into the pores with higher permeability. This would greatly increase the swept volume, thereby increasing the recovery of residual oils. However, the mechanism of foam flooding is not trivial, as it deals with properties of rocks, oil and foams. A central topic is how to enhance the regulation function of foams under external fields, to realize the goal of enhancement of oil recovery (EOR). From the viewpoint of mechanics, the deformation and motion of foams under these input energy is still lacking, and a lot of related work is ongoing.

A great deal of efforts have been made to explore the physical and chemical properties of foams, via experiments and numerical simulations. Raza^4^ carried out the experiment to study the factors affecting the foam performance in porous media. He found that the type of foaming agent and the concentration of foaming agent solution, mainly determine the physical characteristics of foams. In order to improve the stability of the foam, Horozov et al.^5^ proposed to add nanoparticles into the foam system. They declared that the nanoparticles could be adsorbed on the gas–liquid interface of the foam, which increases the flow resistance of the liquid and thus slows the drainage of the liquid film. Based on this idea, Sun et al.^6,7^ prepared a foam system composed of silica nanoparticles and sodium dodecyl sulfate, and found that these two components have a good synergistic foam stabilization effect. Sethrmadhavan et al.^8^ verified this conclusion through a series of numerical simulations. They also drew the conclusion that the nanoparticles could effectively reduce the thinning rate of liquid film and significantly improve the stability of foam system. It should be mentioned that, most of these studies only give some qualitative results, and a quantitative description of foam flooding process has rarely been reported. Another question is that, merely exerting the pressure field is not powerful enough to realize the efficient propulsion of oils in rocks. The reason is that, the influence area of foam fluids under the action of gravity and capillary force is very limited, which cannot lead to an excellent effect on the oil displacement efficiency.

Many experiments show that, the overall control of foam flooding by external fields can effectively increase its swept area, which is of great significance to the evolution of foam movement and deformation in pore channels. Among others, the magnetic field is expected to be used in foam flooding, which lies in its advantages of non-contact control, high degree of freedom, convenient design, flexible use, etc. Besides adopted in petroleum engineering, the magnetic field has huge application prospects in many other engineering fields, such as drug delivery^9^, micro-device control^10^ non-destructive flaw detection^11^ and minimally invasive surgery^12,13^. For example, in the field of magnetic robots, the three-dimensional magnetization printing technology has been applied to prepare soft robots capable of complex shape changes, including deformable soft electronic devices, soft micro robots that can jump, crawl, roll, and transport drug doses^14–17^. In microfluidics, Zhao et al.^18^ used ferromagnetic micro-nanoparticles to prepare liquid marbles or particle rafts to obtain a magnetic field responsive intelligent material to control its movement and deformation through magnetic field loading. This phenomenon greatly enhances the possibility of real-time manipulation of micro-fluidics. Legchenkova et al.^19^ used a stable magnetic field to drive millimeter soap and glycerin bubbles floating on the liquid. They declared that the role of gravity in the displacement of a floating diamagnetic object driven by a stable magnetic field is negligible, and this gives a partial explanation for the movement mechanism of the bubble under the action of the magnetic field. Roy et al.^20^ proposed a diamagnetic droplet movement model driven by a permanent magnetic field, and the associated movement of silicone oil bubbles was studied. Moreover, Borglin et al.^21^ simulated the Ferro fluid in a two-dimensional sand-filled slab in the laboratory. The result shows that the ferromagnetic fluid flows along the direction of the magnet under the action of magnetic force. In addition, as the distance from the magnet gets closer, the magnetic force becomes larger and the flow velocity increases. Hertshorne et al.^22^ used microfluidic chips as visualized micro-scaled porous media to study the migration of magnetic fluids in pores, and the magnets were used to control the flow process. Chai et al.^23^ used the Ferro fluid slug in the microchannel as a kind of thruster to drive the front-end fluid flow under the action of an external magnetic field, and its function is similar to that of a micro-pump. Furthermore, Huang et al.^24^ analyzed the mechanism of ferromagnetic fluid flooding to enhance the oil recovery by a two-dimensional heterogeneous and fractured sand filling plate model, compared with the traditional water flooding. Although a plethora of work has been done on crude oil exploration by magnetic fluids, there remain some shortcomings for these fluids, such as poor sealing effect and weak oil washing ability, which affect their wider applications. Therefore, new displacement media with a strong oil displacement ability, high stability, and sensitive controllability are highly demanded.

Based on previous studies related with foams and magnetic fields, we propose a strategy to add magnetic particles into the mature foam system, and then the foam’s motion can be regulated under the action of magnetic field. This study is directed towards to explore the mechanism of enhancing the oil recovery with the aid of foams, especially in the underground porous rocks. The outline of this article is laid out as follows. In “Experiment” section, the experimental materials and methods are introduced. In “Results and discussion” section, the half-life of the foam after adding magnetic particle is measured, the distribution of magnetic force on the magnetic particles is characterized, and the process of foam movement is described. The effects of four variables on the maximum displacement and maximum accelerations of the magnetic foam are investigated, including the volume of the foam, the volume of the oil droplet, the mass concentration of magnetic particles and the wettability of the substrate. The experimental results have been partially compared with the theoretical modeling, i.e. the dimensional analysis results. Finally, main conclusions follow in “Conclusions” section.

## Experiment

### Preparation of foams

The material of the nanoparticle is the fumed silica, which is purchased from the Evonik Specialty Chemicals Co., Ltd. (China). The original diameter of the silica particle is 20 nm approximately. The Young’s contact angle of water on the original SiO_2_ nanoparticle material is 141.5° and that on the magnetic nanoparticle material is 126°, and these values are provided by the corporation. The Sodium Dodecyl Sulfate (SDS) with a purity of 92.5–99.9% is purchased from the McLean Biochemical Technology Co., Ltd. The deionized water is used in all solutions.

The surfactant solution (SDS) with the volume of 3 ml is poured into the ultrapure water with the volume of 97 ml, and then the mixture is stirred clockwise with a glass rod for 30 s to prepare the surfactant solution with the concentration of 3%. Next, SiO_2_ nanoparticles with the mass of 1 g and magnetic particles with different mass concentrations are added into the surfactant solution, and then we stir the mixture at a constant speed of 8000 r/min for 60 s to prepare the magnetic foam.

In order to characterize the performance of the magnetic foam, let the mixed magnetic foam stand still for 2 min to evaporate. The purpose is to break the large bubbles generated on the surface, which are caused by the uneven stirring process. We draw the magnetic foam with the volume of 2 ml by a syringe, where the syringe is 2 cm away from the foam surface. In succession, we pour the prepared magnetic foam into a wide-range measurement cylinder and put it into the measurement cylinder to record its half-life *t*_50%_.

### Foam movement carrying oil droplet driven by the magnet

**Figure 1:**
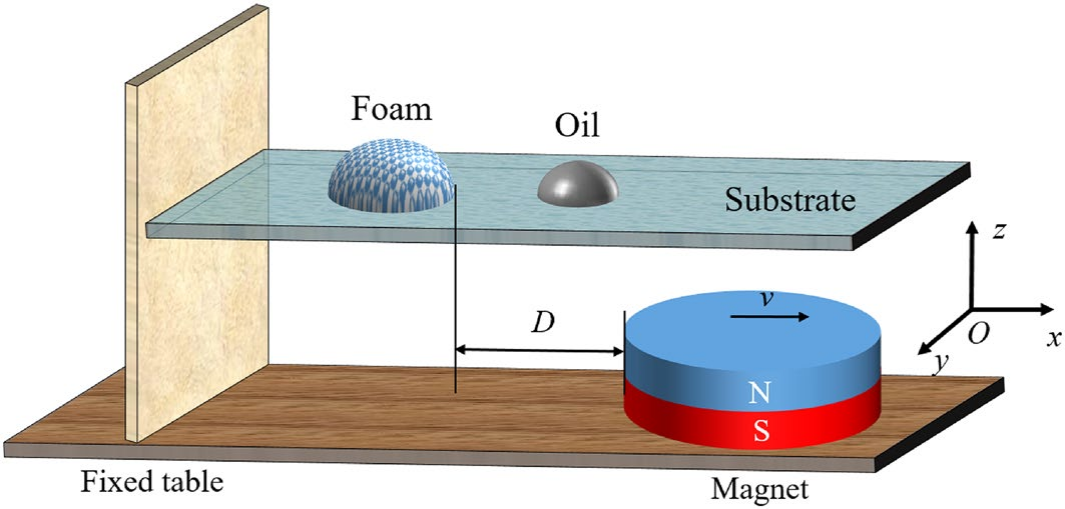
Schematic diagram of the experimental setup.

As is shown in Fig. 1, a cylindrical magnet with a diameter of 90 mm is placed horizontally on the experimental table, and the base is connected to the mobile table. In the experiment, we keep the distance between the foam and the magnet as a constant, to ensure that the magnetic force exerted on the foam is invariable. We place the foam and oil droplet on the substrate, and the horizontal distance between the foam and the magnet is kept as a constant *D* = 10 mm. The magnetic field is applied on the foam with the duration time *T* = 20 s. It can be observed that, when the magnet moves with a constant speed, the magnetic foam moves directionally under the action of the magnetic force. The maximum displacement of the foam is designated as *x*, and the maximum acceleration of the foam is denoted by *a*.

In the experiment, we consider the following physical parameters: the volume of the foam *V*_F_, the volume of the oil droplet *V*_O_, the mass concentration of the magnetic particles *C*_P_, and the Young’s contact angle of surfactant $\theta_{{\text{Y}}}$. The effects of the above factors on the maximum displacement and maximum acceleration of the foam in the motion are analyzed. The viscosity of oil is measured as $\mu_{{\text{O}}}$ = 100 mPa·s, and the density of foam is $\rho$ = 0.8 g/cm^3^.

The effects of the volume of the foam, the volume of the oil droplet, the mass concentration of magnetic particles and the Young’s contact angle of surfactant on the maximum displacement and maximum acceleration of the foam are thoroughly probed in the experiment. Firstly, when considering the influence of the foam volume, we keep the values *V*_O_ = 1.5 cm^3^, *C*_P_ = 2% and $\theta_{{\text{Y}}}$ = 62 ± 0.3°. Then, the displacement and acceleration of the magnetic foam with different volumes of *V*_F_ = 0.5, 1.0, 1.5, 2.0 and 2.5 cm^3^ are recorded respectively. Next, with other variables unchanged and the value of *V*_F_ = 2 cm^3^, six different oil droplets with the volumes of *V*_O_ = 0.3, 0.5, 0.8, 1, and 1.2 cm^3^ are investigated. Thirdly, the magnetic nanoparticles with different concentrations of *C*_P_ = 1.0%, 1.5%, 2.0%, 2.5%, 3.0%, and 3.5% are explored, where the parameter values of other variables remain unchanged. At last, we use three substrates with different wettability, i.e. the acrylic plate, the glass plate, and the PTFE plate in the experiments. The surface of the substrate is smooth enough and the influence of friction is ignored during the experiment. The profile and contact angles of a liquid droplet on the solid substrate are captured by the contact angle goniometer (Biolin Scientific Corporation, Thetalit 100). The Young’s contact angles of the surfactant droplet deposited on the glass plate is $\theta_{{\text{Y}}}$ = 62 ± 0.3°, that on the acrylic plate is $\theta_{{\text{Y}}}$ = 86 ± 0.8°, and that on the PTFE plate is 126 ± 0.7°.

## Results and discussion

### Foam performance evaluation

**Figure 2:**
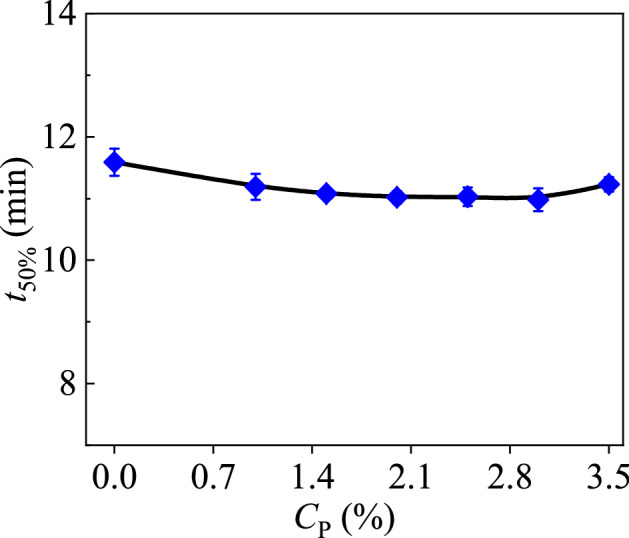
The function curve of the half-life of the foam with respect to the concentration of the magnetic particles.

It should be noted that, the foam system is thermodynamically unstable, and how to form a stable foam is one central task to improve the oil recovery. The effect of nanoparticles on the stability of foam system is usually characterized by measuring the half-life of the foam *t*_50%_. In this way, the influence of the particle concentration, surfactants, and the external environment on the foam stability has been already investigated. In the experiment, we only pay attention to the effect of magnetic particles on the foam stability, where the concentration of SDS solution is kept at 3% and that of the SiO_2_ nanoparticles is kept at 1%. In the experiment, six types of magnetic particles with different concentrations are studied in order to study the foam stability, i.e. *C*_P_ = 0%, 1%, 1.5%, 2%, 2.5%, 3% and 3.5%, where the half-life of the foam is measured. As shown in Fig. 2, the half-life of the foam does not change significantly with the increase of the magnetic particles, and the value is almost *t*_50%_ = 10 min. This fact indicates that the surfactant solution and nanoparticles has reached saturation, and further addition of magnetic particles does not affect the foam system. During this situation, the entire system has arrived at a stable state, as the added nanoparticles only exist on the skeleton connected by the foam bubbles. This behavior can ensure the foam possesses a certain degree of stability, and then the prepared foam can respond quickly when subjected to the magnetic force. This experiment shows that nanoparticles play an essential role on the foam stability, as they increase the strength of the liquid film, slow down the discharge cycle, and help increase the value of *t*_50%_^25–27^.

### Magnetic field description

We then characterize the magnetic force applied on the magnetic particle. In the analysis, all the units we used are SI system units. The magnetic force of a unit mass of magnetic particles *F*_0_ is correlated with the vacuum permeability *µ*_0_, the magnetic susceptibility of magnetic particles *χ* and the magnetic field strength ***H***, with the expression of

1 $$F_{0} = \mu_{0} \chi {\varvec{H}} \cdot \nabla {\varvec{H}},$$

where *µ*_0_ = 4π × 10^−7^ Tm/A. The value of ${\varvec{H}} \cdot \nabla {\varvec{H}}$ can be expanded in terms of the matrix in the Cartesian coordinate system *O-xyz*:

2 $$\left[ {{\varvec{H}} \cdot \nabla {\varvec{H}}} \right] = \left[ {\begin{array}{*{20}l} {H_{x} \frac{{\partial H_{x} }}{\partial x} + H_{y} \frac{{\partial H_{y} }}{\partial x} + H_{z} \frac{{\partial H_{z} }}{\partial x}} \hfill \\ {H_{x} \frac{{\partial H_{x} }}{\partial y} + H_{y} \frac{{\partial H_{y} }}{\partial y} + H_{z} \frac{{\partial H_{z} }}{\partial y}} \hfill \\ {H_{x} \frac{{\partial H_{x} }}{\partial z} + H_{y} \frac{{\partial H_{y} }}{\partial z} + H_{z} \frac{{\partial H_{z} }}{\partial z}} \hfill \\ \end{array} } \right].$$

The magnetic field strength is expressed as *H* = *H*_*x*_***i*** + *H*_*y*_***j*** + *H*_*z*_***k***, where ***i***, ***j*** and ***k*** are three unit vectors along *x*, *y* and *z* directions, respectively.

**Figure 3:**
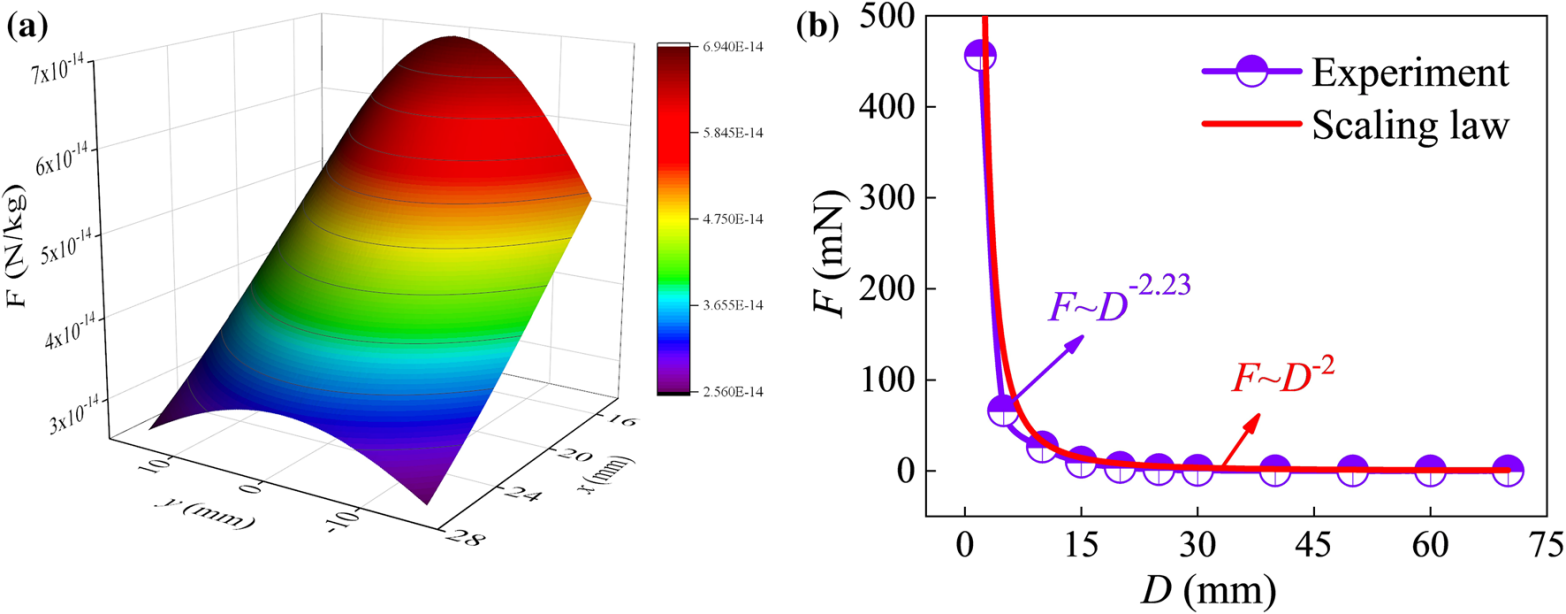
Magnetic force distribution map. (**a**) The cloud map of magnetic force per unit mass of magnetic particles, (**b**) The relationship between magnetic force and the distance *D*.

The Cartesian coordinate system is shown in Fig. 1, and the magnetic field can be computed through the magnetic field module of COMSOL (COMSOL Multiphysics), which is demonstrated in Fig. 3a. Therefore, we can get the values of the three components of the magnetic force in three directions *x*, *y* and *z*. Evidently, the magnetic force in the *x* direction provides the driving force to propel the foam; in the *y* direction it causes the foam to move perpendicular to the direction of movement; and in the *z* direction it causes the deformation of the foam. As the effect of the magnetic force in the *y* direction is minute, it has been omitted in the following analysis.

It is observed in the experiment that, the foam collapses along *z* direction, and this phenomenon can be easily explained that the magnet generates a compressive force, and in the *x* direction the magnet produces a driving force to push the foam to move.

We then get the relationship between the magnetic force of magnetic particles with a unit mass *F*_0_ and the distance *D* through experiments. It is assumed that the magnetic particles are uniformly distributed in the foam during the foam generation process, the magnetic force applied on the foam can be expressed as *F* = *F*_0_*m*_0_, where *m*_0_ is the mass of all the magnetic particles distributed in the foam. Then the relationship between the magnetic force *F* and the distance *D* can be obtained, which is shown in Fig. 3b. The curve shows that as the distance increases, the magnetic force drops rapidly. For example, when *D* > 20 mm, the magnetic force is close to zero. By fitting the experimental data in Fig. 3b, we get the relation that *F* ~ *D*^−2.23^.

It is judged that the magnetic force *F* is mainly related to the distance *D* between the foam and the magnet, the volume of the foam *V*_F_, the concentration of magnetic particles *C*_P_, the density of foam $\rho$, the magnetic field action time *T* and the contact angle between the surfactant solution and substrate $\theta_{{\text{Y}}}$. The following expression can be made according to the dimensional analysis:

3 $$F \sim \frac{{\rho V_{{\text{F}}}^{2} }}{{D^{2} T^{2} }}C_{{\text{P}}}^{{b_{1} }} \theta_{{\text{Y}}}^{{b_{2} }} ,$$

where *b*_*i*_ (*i* = 1, 2, 3, …) is the power index of a physical quantity.

From the theoretical aspect, when the other parameters are fixed, Eq. (3) reduces to *F* ~ *D*^−2^. It is seen that, the values of − 2.23 and − 2 are very close, which indicates the experimental result is reliable.

### Magnetic foam moving process

**Figure 4:**
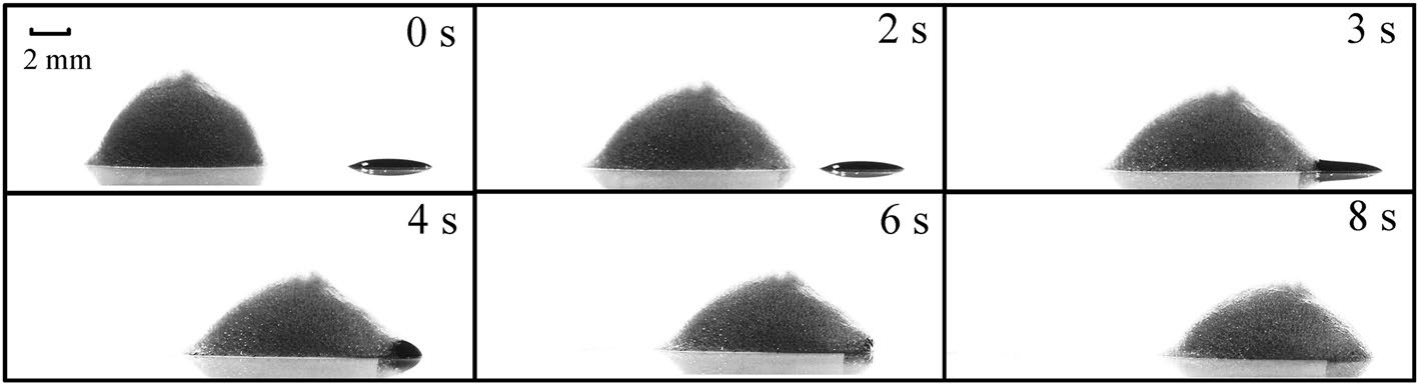
Snapshots of the magnetic foam option process. When *t* = 2 s, the foam starts to move; when *t* = 3 s, the foam comes into contact with oil droplet; and when *t* = 8 s, the foam starts to move with the oil droplet.

In the experiment, the snapshots of the magnetic foam at three stages are recorded, i.e. onset, interplay with oil droplet, and movement by carrying oils, which are shown in Fig. 4. Firstly, the end of the foam near the magnet is activated to move owing to the force of the magnetic field, with the distance *D* = 10 mm. Then under the action of the magnetic field, the foam is accelerated forward. When the elapsed time *t* = 3 s, the surfactant in the foam interacts with the oil droplet. As a result, the surface tension of the oil droplet in the acting part is suddenly reduced, and then the oil droplet cannot maintain the original stable state and become collapsed. When the time *t* = 6 s, more foams wrap the oil droplet to facilitate the emulsification of oil droplet. Next, when *t* = 8 s, the foam gradually emulsifies the oil droplet, and the oil droplet becomes one part of the whole foam–oil system. After that, the magnetic foam continues to move by carrying the oil under the magnetic force.

During the moving process of the foam, due to the adhesion force between the oil droplets and the substrate, the resistance of the foam–oil system gradually increases. When the time *t* = 12 s, the foam stops moving attributed to the resistance. Besides the adhesion between the oil and the substrate hindering the foam, it is observed that with the interaction between the surfactant in the foam and the oil film, some bubbles inside the foam may lose water and burst. It is also noticed that, part of the magnetic particles fall on the bottom of the foam under the influence of gravity. As a result, the regulating ability of the magnetic particles on the foam is greatly weakened, and finally the foam stops moving as the resistance of the substrate is bigger than the driving force of the magnetic field.

### Maximum displacement of the foam

**Figure 5:**
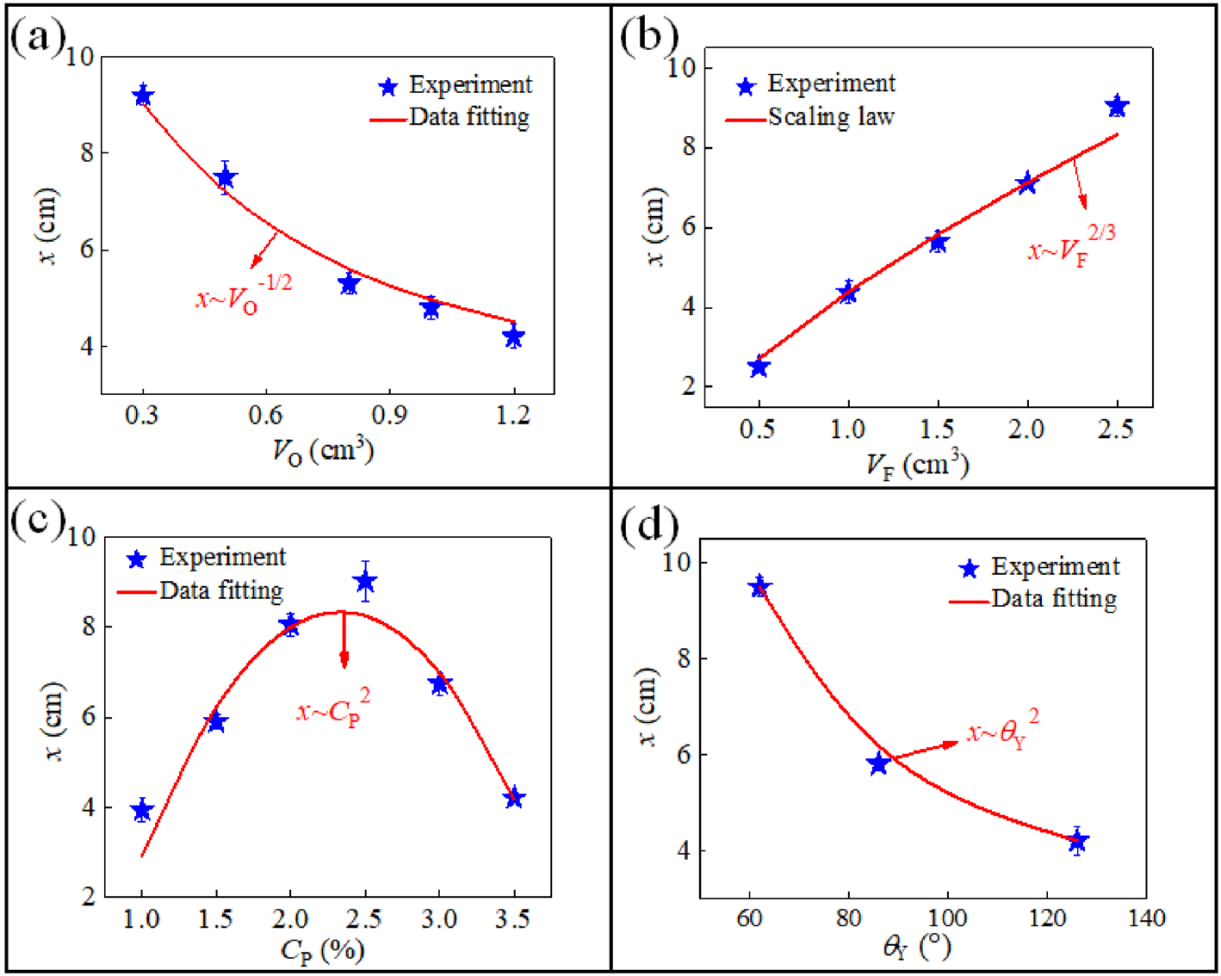
Dependence relationship between the maximum displacement *x* and various parameters, i.e. (**a**) the volume of the oil drop *V*_O_, (**b**) the volume of the foam *V*_F_, (**c**) the concentration of the magnetic particles *C*_P_, and (**d**) the Young's contact angle $\theta_{{\text{Y}}}$.

The oil displacement ability of magnetic foam is mainly characterized by the maximum displacement *x* and maximum acceleration *a* during the foam movement. Firstly, the influence of each variable mentioned above on the maximum displacement of the movement is explored by both experiments and theoretical analyses. Due to the complexity of the system, the dimensional analysis is made to discuss the above experimental results. The maximum displacement of the foam *x* is related with many factors, which mainly include the distance *D*, the volume of the oil droplet *V*_O_, the Reynolds number Re, the concentration of magnetic particles *C*_P_, the contact angle between surfactant solution and substrate $\theta_{{\text{Y}}}$. As a consequence, the scaling law can be obtained as

4 $$x \sim \sqrt {\frac{{D^{5} }}{{V_{{\text{O}}} }}} {\text{Re}}^{2} C_{{\text{P}}}^{{b_{4} }} \theta_{{\text{Y}}}^{{b_{5} }} ,$$

5 $${\text{Re}} = \frac{{\rho v\sqrt[3]{{V_{F} }}}}{\mu },$$

where $\mu$ is the viscosity of the oil, and *v* is the velocity of the foam. This scaling law can be definitely used to analyze the obtained experimental results. First, the dependence relation between the volume of oil droplet *V*_O_ and the maximum displacement of the foam *x* is exhibited in Fig. 5a. It can be seen that, with the increase of *V*_O_, the value of *x* gradually decreases. Especially, when the value of *V*_O_ is 0.3 cm^3^, the maximum displacement of the foam reaches 9.1 cm. This phenomenon can be elucidated that, the smaller the oil drop’s volume is, the smaller its adhesion area on the substrate is, and the smaller the resistant force is. Moreover, by fitting the experimental data in Fig. 5a, we get the relation that *x* ~ *V*_O_^−0.55^. This power law relation can be verified by the dimensional analysis result given in Eq. (4). When the parameters *D*, *V*_F_, *C*_P_, and $\theta_{{\text{Y}}}$ are fixed, Eq. (4) reduces to *x* ~ *V*_O_^−1/2^. It can be seen that, the power values of − 0.55 and − 1/2 are very close, which indicates that the experimental result is consistent with that derived by the dimensional analysis, and thus the experiment is reliable. Obviously, the volume of the oil droplet is negatively related to the maximum displacement of the foam. In the petroleum engineering, too many oils with high-viscosity would greatly increase the adhesion strength with the reservoir. Thus increasing the concentration of the displacement fluid or increasing the external field strength is more conducive to reduce the adhesion of oils inside rocks, aiming to improve the oil recovery.

Next, the relation between the volume of the foam *V*_F_ and the maximum displacement *x* is displayed in Fig. 5b. The curve shows that, with the increase of *V*_F_, the value of *x* gradually increases. It is because that, when the volume of the foam is bigger, it includes more magnetic particles and induces a bigger driving force. In addition, with a bigger volume, the foam can more effectively envelop the oil droplet, and thus it can easily carry the oils. Similarly, by fitting the experimental data in Fig. 5b, we get the relation that *x* ~ *V*_F_^0.698^. When the parameters *D*, *V*_O_, *C*_P_, and $\theta_{{\text{Y}}}$ are fixed, the scaling law in Eq. (4) reduces to *x* ~ *V*_F_^2/3^. It can be seen that, the values of 0.698 and 2/3 do not differ much, which indicates that the prediction of theoretical analysis is in good agreement with the experimental data. It is obvious to find that, the volume of the foam has a great influence on the displacement effect. However, to keep the effectiveness of the foam is a kernel issue and it deals with the stability of the foam. In practice, the liquid film drainage will make the film thinner, and the thinner liquid film will eventually produce a cavity. As time goes by, the cavity gradually expands causing the liquid film to gradually collapse and disappear, and finally causing the coalescence of bubbles. To form such an effective cavity in the liquid film, an energy barrier must be overcome, which determines the speed of bubble coalescence^28^. The spatial network structure formed by the interaction between the magnetic particles greatly increases the energy barrier. Altogether, the nanoparticles can weaken the drainage of the liquid film, and ultimately reduce the speed of the bubble coalescence, thereby greatly enhancing the stability of the foam.

However, although the scaling law can determine some power values and coefficient values, its function is very limited in some cases, especially when there are some dimensionless variables in the equation, such as the particle concentration and the Young’s contact angle. For instance, the dependence relation between the mass concentration of magnetic particles *C*_P_ and the maximum displacement of the foam *x* is shown in Fig. 5c. It can be seen that, with the increase of *C*_P_, the value of *x* increases first and then decreases, i.e. the curve is not monotonic. Especially, when the value of *C*_P_ is equal to 2.5%, the maximum displacement arrives at the extreme value *x*_max_ = 9.0 cm. When *C*_P_ < 2.5%, the more magnetic particles are dispersed in the foam, the stronger the magnetic force of the foam becomes. However, with a further increase of *C*_P_, part of the magnetic powders inside the foam will sink on the bottom of the foam by gravity, and the magnetic particles fail to propel the foam. Then the relationship between *x* and *C*_P_ can be expressed as $x = {\text{ A}}_{1} C_{{\text{P}}}^{{b_{3} }}$, and the power value *b*_3_ cannot be given via the scaling law as *C*_P_ is a non-dimensional parameter. The coefficient A_*i*_ (*i* = 1, 2, 3, …) is used throughout this article. By fitting the experimental data, we get that *b*_3_ = 2 and A_1_ =  − 3.05 m. This behavior indicates that, there is an optimal choice for the magnetic particle concentration during oil displacement, which is beneficial to guide the practical technologies. It is easy to know that in the foam system in the absence of magnetic particles, the channels among bubbles are narrow. In this case multiple bubbles are closely connected, and the liquid film is pulled tightly under the action of surface tension. The added magnetic particles are mainly distributed in the Plateau channels among bubbles. With the increase of *C*_P_, the Plateau channels in the foam are widely occupied, and the bubble diameter grows smaller. When *C*_P_ = 2.5%, most of the bubbles remain spherical or ellipsoidal, and the stability of the foam reaches the best.

Finally, the maximum displacement of the foam *x* is also a function with respect to the Young’s contact angle between the surfactant solution and substrate $\theta_{{\text{Y}}}$, which is shown in Fig. 5d. The curve shows that, with the increase of $\theta_{{\text{Y}}}$, the maximum displacement goes on decreasing. This phenomenon can be explained that, the foam on a hydrophilic substrate is easier to form a lubricating film (precursor film) on the substrate surface, so that the oil droplet is completely emulsified and the foam bears a smaller resistance. On the contrary, the hydrophobic substrate can provide a larger resistant force and it will cause a smaller displacement. With the other parameters fixed, Eq. (4) reduces to $x = {\text{A}}_{2} \theta_{{\text{Y}}}^{{b_{4} }}$, and the undetermined parameters can be deduced by fitting the experimental data. The result shows that, the maximum displacement *x* is a quadratic function with respect to the Young’s contact angle, that is, $x = 0.5\theta_{{\text{Y}}}^{2}$.

### Maximum acceleration of the foam

Another characterization of the foam’s oil displacement ability is the magnitude of the magnetic force, where we characterize it by using the acceleration. The moving process of the foam can be divided into two stages, i.e. firstly the foam moves alone, and secondly it moves by carrying oils. In stage 1, the experimental base is relatively smooth, so the frictional resistance of the base is ignored during the movement of the foam. In this period, the acceleration of the foam is a constant, and it is variable in the second stage.

As mentioned above, the maximum accelerations of the foam in the two stages are different, which can be expressed as *a*_1_ and *a*_2_, respectively. In the light of the Newton’s second law, the acceleration *a*_1_ can be obtained as

6 $$a_{1} = \frac{F}{m} \sim \frac{{V_{{\text{F}}} }}{{D^{2} T^{2} }}C_{P}^{{b_{5} }} \theta_{{\text{Y}}}^{{b_{6} }} ,$$

where *m* is the mass of the foam, and Eq. (3) is inserted for derivation.

**Figure 6:**
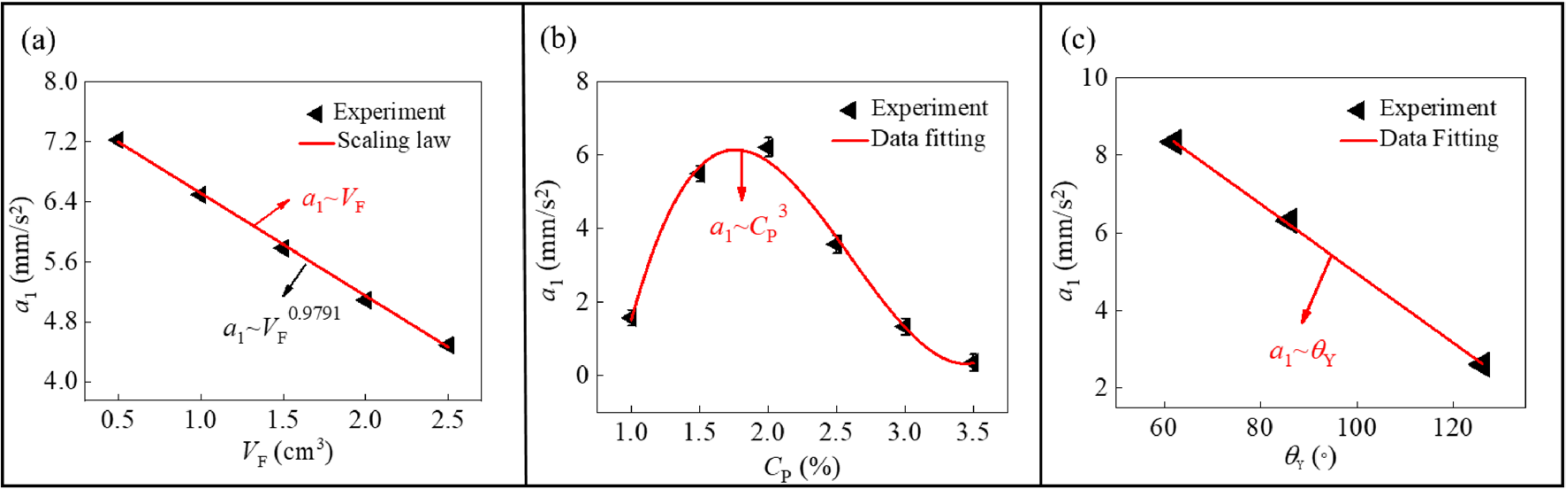
Dependence relationship between the maximum acceleration in the first stage *a*_1_ and various parameters, i.e. (**a**) the volume of the foam *V*_F_, (**b**) the concentration of the magnetic particles *C*_P_, and (**c**) the Young's contact angle $\theta_{{\text{Y}}}$.

We then verify the above scaling law on the basis of a series of experimental data. Firstly, the dependence relation between the foam’s volume and the maximum acceleration of the foam is shown in Fig. 6a. It can be seen that, the value of *a*_1_ gradually decreases with the increase of *V*_F_. Especially, when the value of *V*_F_ is equal to 0.5 cm^3^, the acceleration of the foam reaches *a*_1_ = 7.2 mm/s^2^. This behavior is related to the regulation capability of magnetic particles on the overall foam. As the volume of the foam increases, the height of the foam will also increase, and the foam can collapse along the height direction as it is subjected to the magnetic force. In this process, part of the work done by the magnetic field on the whole foam is transformed into the foam's own deformation energy and is lost, and as a consequence, the magnetic particles lose the capability to quickly drive the whole foam. By fitting the experimental data in Fig. 6a, we get the relation that *a*_1_ ~ *V*_F_^0.9791^. From the theoretical aspect, when the parameters *D*, *T*, *C*_P_, and $\theta_{{\text{Y}}}$ are fixed, Eq. (5) reduces to *a*_1_ ~ *V*_F_. It is seen that, the values of 0.9791 and 1 matches very well, which indicates that the experimental result is reliable.

Next, as shown in the non-monotonic curve in Fig. 6b, with the increase of *C*_P_, the acceleration first increases and then decreases. That is, there is an optimal particle concentration to arrive at an extreme acceleration for the foam, i.e. *C*_P_ = 1.85%. When *C*_P_ < 1.85%, the overall foam is modulated by the magnetic field, and its acceleration becomes bigger with the increase of *C*_P_. However, the magnetic particles with a high value of *C*_P_ will coalesce in the foam, which will sink on the bottom of the foam under gravity, and this action will prevent the motion of the foam. Moreover, when the remaining parameters such as *D*, *V*_F_, *T* and $\theta_{{\text{Y}}}$ are fixed, the relationship between *a*_1_ and *C*_P_ can be estimated as an exponential curve, that is, *a*_1_ ~ A_3_*C*_P_^*b*5^*.* As mentioned above, the power and coefficient in this equation can only be given based on the data fitting from the experimental dots of Fig. 6b, that is, *b*_5_ = 3, and the coefficient A_3_ can be given as 2.44 m/s^2^.

Similarly, the relation between $\theta_{{\text{Y}}}$ and *a*_1_ is displayed in Fig. 6c, where the value of *a*_1_ decreases linearly with the increase of $\theta_{{\text{Y}}}$. Especially, when the value of $\theta_{{\text{Y}}}$ is equal to 62°, the value of *a*_1_ is 8.3 mm/s^2^. The result shows that the maximum acceleration of the foam on the hydrophobic substrate is much smaller than that on the hydrophilic substrate. As shown in the figure, the maximum acceleration of the foam on the hydrophilic substrate is about 4 times that of the hydrophobic substrate. This law can also be explained by the precursor film around the foam as mentioned above. By fitting the experimental data, we get the relation between *a*_1_ and $\theta_{{\text{Y}}}$, which is written as *a*_1_ ~ A_4_*θ*_Y_^*b*6^. As shown in Fig. 6c, *a*_1_ and $\theta_{{\text{Y}}}$ satisfy a linear function relationship, that is, *b*_6_ = 1, and the coefficient A_4_ can be given as − 0.009 m/s^2^.

In stage 2, when the foam is in contact with the oil droplet, the foam’s speed becomes zero. Subsequently, the surfactant in the foam interacts with the oil droplets, causing the rapid decrease of the surface tension of the newly contacted oil film, and the foam continues to move forward under the magnetic force until the oil droplet is completely wrapped. In this situation, the system including foam and oil droplet is subject to the combined action of magnetic field force and adhesion resistance, and the acceleration of the foam–oil system gradually decreases. The maximum acceleration of this process satisfies the Newton’s second law *a*_2_ = *F*ʹ/*m*, where $F^{\prime }$ is the resultant force on the foam–oil system, including the magnetic force and the resistant force of the substrate. The resultant force is correlated with many factors, which mainly deal with the volume of the foam *V*_F_, the density of the foam $\rho$, the capillary numbers *C*_*a*_, the magnetic field action time *T*, the volume of oil *V*_O_, the distance *D*, the concentration of magnetic particles *C*_P_, and the contact angle between surfactant solution and substrate $\theta_{{\text{Y}}}$. Through the dimensional analysis, we can get the following expression:

7 $$F^{\prime } \sim \frac{{C_{a} }}{{D^{2} T^{2} }}\frac{{\rho V_{{\text{F}}}^{3} }}{{V_{{\text{O}}} }}C_{{\text{P}}}^{{b_{7} }} \theta_{{\text{Y}}}^{{b_{8} }} ,$$

8 $$C_{a} = \frac{{\mu_{{\text{O}}} v}}{\gamma },$$

where $\gamma$ is the surface tension of oil. According to the Newton's second law, one can get the expression of the acceleration

9 $$a_{2} = \frac{{F^{\prime } }}{m} \sim \frac{{C_{a} }}{{D^{2} T^{2} }}\frac{{V_{{\text{F}}}^{2} }}{{V_{{\text{O}}} }}C_{{\text{P}}}^{{b_{9} }} \theta_{{\text{Y}}}^{{b_{10} }} .$$

**Figure 7:**
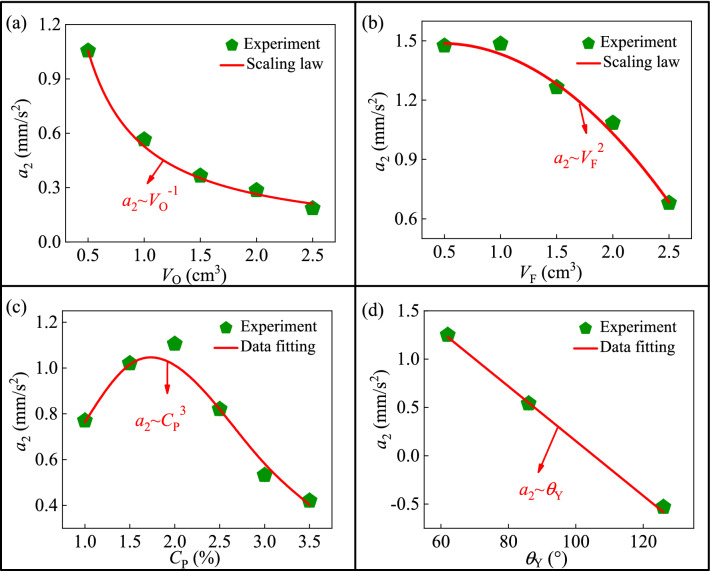
Dependence relationship between the maximum acceleration in the second stage *a*_2_ and various parameters, i.e. (**a**) the volume of the oil drop volume *V*_O_, (**b**) the volume of the foam *V*_F_, (**c**) the concentration of the magnetic particles *C*_P_, and (**d**) the Young's contact angle $\theta_{{\text{Y}}}$.

Equation (9) can be naturally utilized to decipher the obtained experimental results. Firstly, the dependence relation between the volume of oil and the maximum acceleration is demonstrated in Fig. 7a. It can be seen that, with the increase of *V*_O_, the value of *a*_2_ persists in decreasing. Especially, there is an extreme value for the maximum acceleration 1.02 mm/s^2^ when the value of *V*_O_ is equal to 0.3 cm^3^. This phenomenon can be explained that, the larger the oil droplet is, the less the foam can completely emulsify the oil droplet, which makes the adhesion force between the bottom of the oil droplet and the substrate bigger, and this would hinder the movement of the foam. Moreover, by fitting the experimental data in Fig. 7a, we get the relation that *a*_2_ ~ *V*_O_^−1.04^. This power law relation can be verified by Eq. (9), which reduces to *a*_2_ ~ *V*_O_^−1^. Evidently, the experimental value − 1.04049 fits the theoretical value − 1 very well, which indicates that the experimental result is consistent with that derived by the dimensional analysis.

Next, the relation between the volume of the foam and the maximum acceleration is displayed in Fig. 7b. With the increase of *V*_F_, the value of *a*_2_ gradually increases, as the influence of magnetic force on the foam decreases. Similarly, by fitting the experimental data in Fig. 7b, we get the relation that *a*_2_ ~ *V*_F_^2.136^. When the other parameters are fixed, Eq. (9) reduces to *a*_2_ ~ *V*_F_^2^ in theory. It can be seen that, the values of 2.136 and 2 are very close, which indicates that the theoretical prediction is in agreement with the experimental data.

Thirdly, the dependence relation between *C*_P_ and *a*_2_ is shown in Fig. 7c, where the value of *a*_2_ increases first and then decreases with the increase of *C*_P_. This indicates that there must be an optimal value for the particle concentration. In particular, when the value of *C*_P_ is 2.0%, the value of *a*_2_ arrives at an extreme value 1.1 mm/s^2^. It is also noted that the acceleration in the second stage is much smaller than that in the first stage. Similarly, the relationship between *a*_2_ and *C*_P_ can be given as *a*_2_ ~ A_5_*C*_P_^*b*9^. By fitting the experimental data, the parameters can be identified as *b*_9_ = 3 and A_5_ = 0.1923.

Finally, as shown in Fig. 7d, the maximum acceleration decreases with the increase of the Young's contact angle between the surfactant solution and substrate. By fitting the experimental data in Fig. 7d, the value of *a*_2_ is expressed as a linear function with respect to the Young’s contact angle if the other parameters are fixed, that is, *a*_2_ ~ A_6_*θ*_Y_^*b*10^, where *b*_10_ = 1 and the coefficient A_6_ is − 0.0283.

## Conclusions

In conclusion, the motion of a foam with magnetic nanoparticles under the action of a magnetic field is comprehensively studied in the present work. Firstly, the half-life of the magnetic foam is measured by varying the concentration of magnetic particles, and the experiment shows that the magnetic particles have little effect on the half-life of the foam. The magnetic force per unit mass of magnetic particles in the magnetic field is measured through experiments. The maximum displacement and maximum acceleration of the foam are fully investigated via experiments and theoretical analyses. The critical factors including the volume of the foam, the volume of the oil droplet, the mass concentration of magnetic particles and the Young’s contact angle of surfactant on solid are surveyed as controllable variables. The function curves of the maximum displacement and the maximum acceleration with respect to these variables are given in the experiment, and some optimal parameter values are advised for selection in engineering. The experimental relations can be partially verified by the scaling laws, and the other values for the power and coefficient can be obtained via data fitting. These findings are beneficial to understand the oil displacement with the aid of magnetic field, which provide some inspirations for drug delivery, robots and micro-fluidics.
